# Integrin β3 Induction Promotes Tubular Cell Senescence and Kidney Fibrosis

**DOI:** 10.3389/fcell.2021.733831

**Published:** 2021-11-05

**Authors:** Shen Li, Song Jiang, Qingyan Zhang, Bo Jin, Daoyuan Lv, Wenju Li, Min Zhao, Chunming Jiang, Chunsun Dai, Zhihong Liu

**Affiliations:** ^1^National Clinical Research Center of Kidney Diseases, Jinling Hospital, Nanjing University Medical School, Nanjing, China; ^2^Department of Nephrology, Nanjing Drum Tower Hospital, Nanjing University Medical School, Nanjing, China; ^3^Center for Kidney Disease, The Second Affiliated Hospital of Nanjing Medical University, Nanjing, China; ^4^Department of Clinical Genetics, The Second Affiliated Hospital of Nanjing Medical University, Nanjing, China

**Keywords:** ITGB3, isoliquiritigenin, renal fibrosis, cell senescence, TGF-β1

## Abstract

Tubular cell senescence is a common biologic process and contributes to the progression of chronic kidney disease (CKD); however, the molecular mechanisms regulating tubular cell senescence are poorly understood. Here, we report that integrin β3 (ITGB3) expression was increased in tubular cells and positively correlated with fibrosis degree in CKD patients. ITGB3 overexpression could induce p53 pathway activation and the secretion of TGF-β, which, in turn, resulted in senescent and profibrotic phenotype change in cultured tubular cells. Moreover, according to the CMAP database, we identified isoliquiritigenin (ISL) as an agent to inhibit ITGB3. ISL treatment could suppress Itgb3 expression, attenuate cellular senescence, and prevent renal fibrosis in mice. These results reveal a crucial role for integrin signaling in cellular senescence, potentially identifying a new therapeutic direction for kidney fibrosis.

## Introduction

Cellular senescence means cells undergoing permanent proliferative arrest with associated changes involving chromatin organization, gene transcription, and protein secretion (Campisi, 2013). In kidney, proximal tubular cell is the major senescent cell type during the chronic injury (Sis et al., 2007). Tubular senescence is presented at high level in many types of renal disease, such as IgA nephropathy and diabetic nephropathy (DN) (Szeto et al., 2005; Verzola et al., 2008; Docherty et al., 2019). Accumulating evidence indicates that the severity of tubular senescence correlates with kidney injury, loss of renal function, and renal fibrosis (Al-Douahji et al., 1999; Wolf et al., 2005; Baker et al., 2016; Baar et al., 2017). Senescent cells drive kidney fibrosis mainly by producing and releasing senescence-associated secretory phenotype (SASP), a collection of proinflammatory and profibrotic factors and cues, such as interleukin-1β (IL-1β), C-X-C motif chemokine ligand 1 (CXCL1), and transforming growth factor-β1 (TGF-β1). SASP forms a detrimental microenvironment that regulates the behavior of neighboring cells (Acosta et al., 2008; Lopez-Otin et al., 2013; Tchkonia et al., 2013). Recently, studies showed that cellular senescence may activate p53, p21, and p16, induce cell cycle arrest, release SASP, and promote renal fibrosis (Yang et al., 2010; Campisi, 2013; Childs et al., 2015). However, the studies about tubular senescence are mainly focused on the intracellular processes; the extracellular signal delivery of senescence is poorly understood in kidney.

Integrins, a family of heterodimeric cell surface receptors, consisted of α and β subunits. Nineteen α and eight β subunits have been identified to date, forming at least 25 αβ heterodimers (Takagi and Springer, 2002). Integrins serve as cellular adhesion providers and downstream signal mediators (Hynes, 2002) that affect numerous cellular processes, including cell adhesion, apoptosis, migration, proliferation, survival, and senescence (Hynes, 2002). Integrin function is also implicated in *Drosophila* senescence (Goddeeris et al., 2003). In cultured human fibroblasts, integrin β1 (ITGB1), more specifically α6β1 integrin–heparin sulfate proteoglycan complexes, mediates cellular communication network factor 1 (CCN1)-induced senescence during wound healing (Jun and Lau, 2010). Recent studies reported that integrin β3 (ITGB3) is significantly elevated in the senescent cells and tissues from aged organisms. It induces senescence *via* p21 and p53, and increases the release of TGFβ in SASP (Rapisarda et al., 2017). These data collectively suggest a role for aberrant integrin signaling in senescence, but how integrin alters tubular senescence and how this contributes to kidney fibrosis deserves further study.

The above consideration led us to search for a mechanistic link between integrin and senescence during kidney injury. We found that ITGB3 was a hub gene in chronic kidney disease (CKD) patients with tubulointerstitial fibrosis. ITGB3 was increased sharply with fibrosis degree. Overexpression of ITGB3 led to p53 activation and entry into senescence and TGF-β secretion in tubular cells. Moreover, targeting ITGB3 with isoliquiritigenin (ISL), an agent screened *via* Connectivity Map (CMAP) database, reduced tubular cell senescence and kidney fibrosis in mouse with unilateral ureteral obstruction (UUO) nephropathy.

## Materials and Methods

### Animal

We performed all animal care and experiments according to the guidelines of the Institutional Animal Care and Use Committee at Jinling Hospital of Nanjing University.

The db/db mice in C57BL/6J background and db/m mice were obtained from the Jackson Laboratory. Body weight and fasting blood glucose levels were monitored weekly.

The UUO model was performed as previously described (Meng et al., 2016). Briefly, we anesthetized C57BL/6J mice (8 weeks, 22–25 g) with intraperitoneal injection of pentobarbital (80 mg/kg). We then divided the mice into two groups: the UUO group and the sham operation group. In the UUO group, we exposed the left ureter *via* a midline incision and ligated it. The sham operation group was handled in a similar manner, but without ureteral ligation. We harvested obstructed kidneys at 7 days after UUO. In a second experiment, UUO mice were dosed intraperitoneally with 30 mg/kg ISL or dimethyl sulfoxide (DMSO) (vehicle) and were euthanized 7 days later. In a third set of experiments, C57BL/6J mice received two tail vein injection of 50 μg His-labeled Itgb3-expressing plasmid DNA (Sino Biological, Beijing, China) or empty vector mixed with *in vivo* jetPEI (PolyPlus) in 5% glucose when UUO model established and 3 days after UUO. Mice were euthanized 7 days later.

The passive Heymann nephritis (PHN) model was established as previously described in our lab (Chen et al., 2010). Briefly, FxIA tubular antigen was prepared from renal cortices of Wistar rats. New Zealand white rabbits were immunized with FxIA antigen and the rabbit antiserum was prepared. Adult female Sprague-Dawley (SEM) rats with body weights of 150–180 g were given two intraperitoneal injections of anti-Fx1A antiserum, 2 and 1 ml sequentially with 1-h intermission. Control and PHN rats were killed at 28 days after injection.

### Plasmid Transfection

For plasmid transfection, cells were seeded to 70–90% confluency at the time of transfection. The cells were transfected with the indicated plasmids using Fugene according to the manufacturer’s protocol (PROGEMA). The transfected cells were collected after 24–48 h.

### Cell Culture and Reagents

HK-2 cells were purchased from the American Type Culture Collection (ATCC) and maintained in F12 medium (GIBCO) with 10% FBS (GIBCO) at 37°C in a 5% CO_2_ humidified incubator.

Pifithrin-α, SB525334, and ISL were purchased from MedChemExpress, reconstituted in DMSO, and used at a final concentration of 20, 1, and 20 μM, respectively. TGF-β1 was purchased from Sigma-Aldrich, reconstituted in PBS and used at a final concentration of 10 ng/ml.

### Transwell Paracrine Assay

For paracrine assays, transwell chambers (Corning, United States) with a membrane pore size of 0.4 μm were utilized. Subsequently, HK-2 cells were seeded in the upper chambers, and ITGB3-overexpressed or empty vector HK-2 cells were seeded in the lower chambers. After incubation for 24 h, the cells in upper chambers were treated with ISL (20 μM) for 24 h.

### Immunohistochemistry and Immunofluorescence Staining

Immunohistochemistry (IHC) was carried out as described previously in paraffin-embedded tissue sections of 2 μm thick (Wu et al., 2015). Primary antibodies were anti-F4/80 (Servicebio), anti-ITGB3 (Santa Cruz), and anti-FN1 (Proteintech). The total number of F4/80-positive cells was quantitated in 10 randomly chosen fields using Image-Pro Plus software. Samples were examined in a blinded manner. Tubular injury was evaluated in Masson section. All the staining was semi-quantified by Image-Pro Plus software.

For immunofluorescence staining, frozen tissue sections were blocked with 1% BSA and incubated in primary antibodies. The sections were then incubated with an FITC-conjugated antibody.

### Transforming Growth Factor-β1 Measurement

The immunologic enzyme-linked immunosorbent assay (ELISA) specific for the active form of TGF-β1 was used to detect the TGF-β1 (BOSTER). Active TGF-β1 was measured directly in untreated samples, whereas total TGF-β1 (active + latent) was measured after pre-treating the samples with 0.2 volume of 1 N HCl for 20 min at room temperature to convert the latent TGF-β1 to active TGF-β1 (Ahamed et al., 2008).

### β-Gal Staining

Cytochemical staining was performed using a senescence β-galactosidase staining kit (Beyotime Biotechnology). The cells or frozen sections were fixed and washed with PBS. After incubation with SA-β-Gal staining solution overnight at 37°C, the cells were rinsed twice with PBS and then subject to microscopic examination.

### Protein–Peptide Docking

3D structures of the ITGB3 protein and RGD peptide were modeled based on X-ray crystal structures of integrin αIIB β3 headpiece and RGD peptide complex (Zhu et al., 2013). We docked RGD peptide into the binding pocket in ITGB3 using Chimera (version 1.15) and Autodock Vina program.

### Statistical Analysis

All the experiments were performed in triplicate and repeated three times. Data are expressed as mean ± SEM, and were analyzed using SPSS or R software. Statistical significance was assessed by a two-tailed Student’s *t*-test or one-way ANOVA with Tukey’s *post-hoc* test, association was assessed by Pearson association. *p* < 0.05 was considered to be statistically significant. The details of transcriptome analysis are provided in Supplementary Method.

## Results

### Identification of Differentially Expressed Genes in Kidneys With Chronic Kidney Disease

**FIGURE 1:**
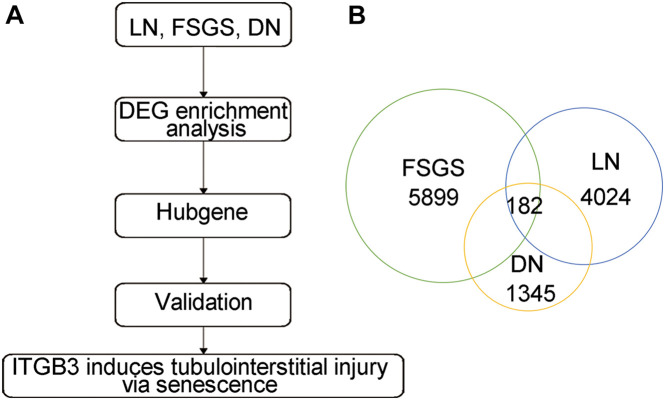
The common DEGs among different types of CKD. **(A)** The strategy of candidate gene screen. **(B)** The common DEGs based on DN, LN, and FSGS.

The gene expression profiles of renal tissues from DN patients (GSE30529), lupus nephritis (LN) patients (GSE112943), and focal segmental glomerulosclerosis (FSGS) patients (GSE99340) were used to explore key genes in CKD (Figure 1A; Woroniecka et al., 2011; Dey-Rao and Sinha, 2015; Shved et al., 2017). By controlling the false discovery rate (FDR), we defined differentially expressed genes (DEGs) as genes with adj. *p*-value < 0.05 and | log2 fold change (FC)| > 1. The common DEGs were used for further analysis. We found 1,345 DEGs in GSE30529, 4,024 DEGs in GSE112943, and 5,899 DEGs in GSE99340; only 182 DEGs were shared in DN, LN, and FSGS patients (Figure 1B and Supplementary Figures 1A–C; for full data, see Supplementary Data 1). It is speculated that these 182 intersecting DEGs were the key regulators in CKD progression.

### Hub Gene Identification

**FIGURE 2:**
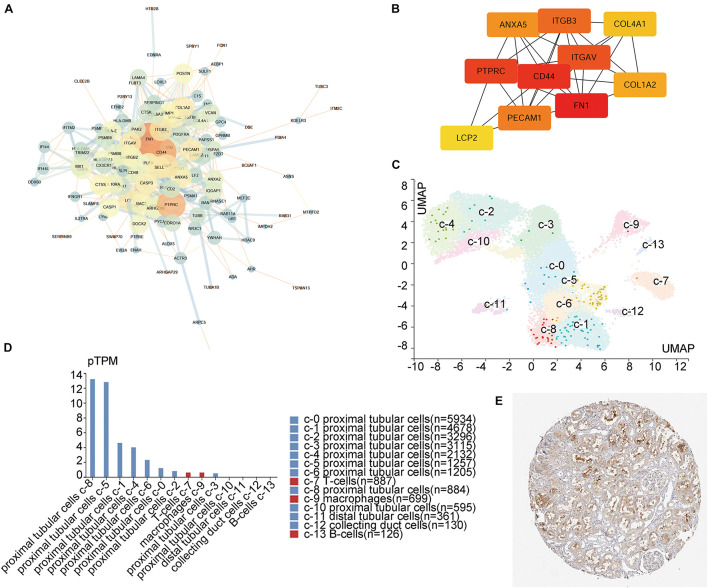
Integrin β3 (ITGB3) is the hub gene in CKD. **(A)** Entire PPI network analysis and **(B)** hub-gene identification of the DEGs. **(C)** Unsupervised scRNA-seq analysis distinguishing 14 different cell clusters in kidney cells from living donor based on the Human Protein Atlas Project. **(D)** Clusters and corresponding cell counts of scRNA-seq. **(E)** ITGB3 mainly expressed on renal tubule.

PPI network analysis for these 182 intersecting DEGs showed the 129 most credible direct interactors (Figure 2A and Supplementary Table 1). We identified 10 hub genes from these 129 interactors *via* CytoHubba plugin in Cytoscape (Figure 2B; Shannon et al., 2003). The 10 hub genes, Fibronectin 1 (FN1), CD44, Protein Tyrosine Phosphatase Receptor Type C (PTPRC), Integrin Subunit Alpha V (ITGAV), ITGB3, Platelet And Endothelial Cell Adhesion Molecule 1 (PECAM1), Annexin A5 (ANXA5), Collagen Type I Alpha 2 Chain (COLA2), Collagen Type IV Alpha 1 Chain (COL4A1), and Lymphocyte Cytosolic Protein 2 (LCP2), were all upregulated. By assessing the FC in diseases, association with eGFR and previous literature, ITGB3 interested us. As a member of integrin family, ITGB3, also known as CD61 or GPIIIA, could respond to the stromal and immune microenvironment and promote cellular senescence in many types of tumor (Rapisarda et al., 2017). Additionally, it had been reported that ITGB3 could enhance TGF-β signaling (Asano et al., 2005; Margadant and Sonnenberg, 2010; Rapisarda et al., 2017). Both senescence and TGF-β signaling played an important role in kidney injury. However, there were rare studies about ITGB3 in kidney, and all of those studies were focused on podocyte (Wu et al., 2015; Lang et al., 2019; Wei et al., 2019). Based on the single-cell RNA-seq and IHC staining of human renal tissues from public database (Human Protein Atlas), ITGB3 was mainly expressed in proximal tubular cells (Figures 2C–E). Thus, it is predicted that the upregulation of ITGB3 in renal tubule may play a crucial role in the progression of kidney injury.

### Induction of Integrin β3 in Tubular Cells of Chronic Kidney Disease

**FIGURE 3:**
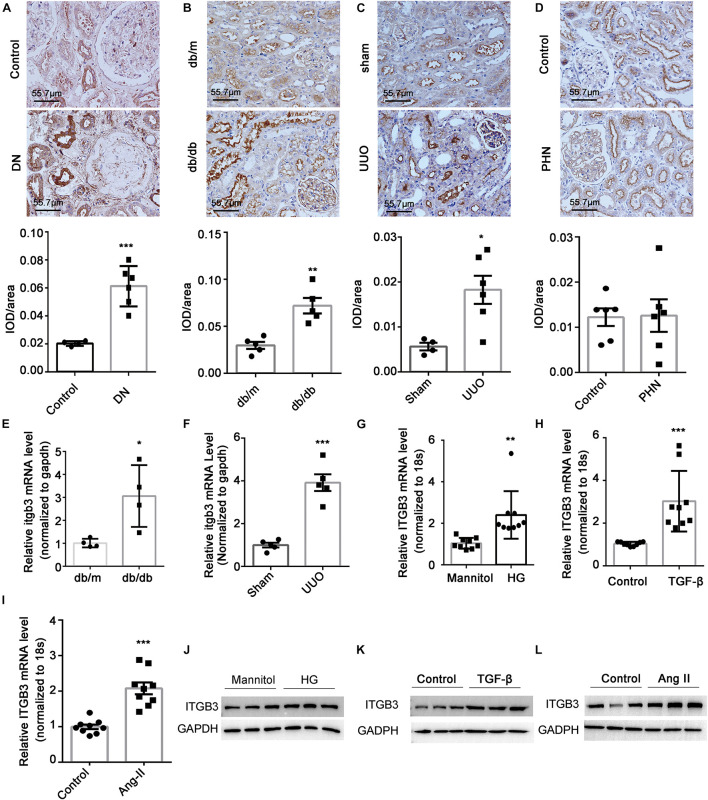
Expression of ITGB3 in tubular tissues in kidney disease. IHC and quantification analysis of ITGB3 in **(A)** patients with DN (*n* = 6 per group) and animal models (*n* = 5 per group) of **(B)** db/db, **(C)** UUO, and **(D)** PHN. The level of Itgb3 mRNA in tubular tissues of **(E)** db/db and **(F)** UUO mice. The level of ITGB3 mRNA in HK-2 cells treated with **(G)** high glucose (30 mM, 24 h), **(H)** TGF-β (10 μg/ml, 24 h), and **(I)** Ang II (1 μM, 24 h). The level of ITGB3 protein in HK-2 cells treated with **(J)** high glucose (30 mM, 48 h), **(K)** TGF-β (10 μg/ml, 48 h), and **(L)** Ang II (1 μM, 48 h). Data shown are representative of three experiments. For statistical analysis, a two-tailed Student’s *t*-test was used for panels **(A–I)**. ***p < 0.001. **p < 0.01. *p < 0.05. Scale bar = 55.7 μm.

Along with the transcriptome data of GSE30529, GSE112943, and GSE99340, we also found an increase in the ITGB3 mRNA level in the tubular tissues of patients with DN, FSGS, or LN in other independent cohorts (Supplementary Figures 2A–C; Woroniecka et al., 2011; Berthier et al., 2012; Na et al., 2015). By contrast, no increase in ITGB3 level was detected in the tubule of patients with minimal tubulointerstitial injury, such as minimal change disease (MCD) (Supplementary Figure 2D) and membranous nephropathy (MN) (Supplementary Figure 2E). IHC staining showed that ITGB3 was no significantly difference between the tubule of normal controls and PHN mice, while in DN patients, db/db mice, and UUO mice, the level of ITGB3 was significantly increased in the injured tubule (Figures 3A–D). PCR analysis also showed that Itgb3 was increased in the tubule of db/db mice and UUO mice (Figures 3E,F). We also treated HK-2 cells with high glucose, TGF-β, and Angiotensin II (Ang II) *in vitro*, all of which increased ITGB3 expression (Figures 3G–L).

### Upregulated Integrin β3 Contributes to Tubular Cell Senescence Through p53 Signaling

**FIGURE 4:**
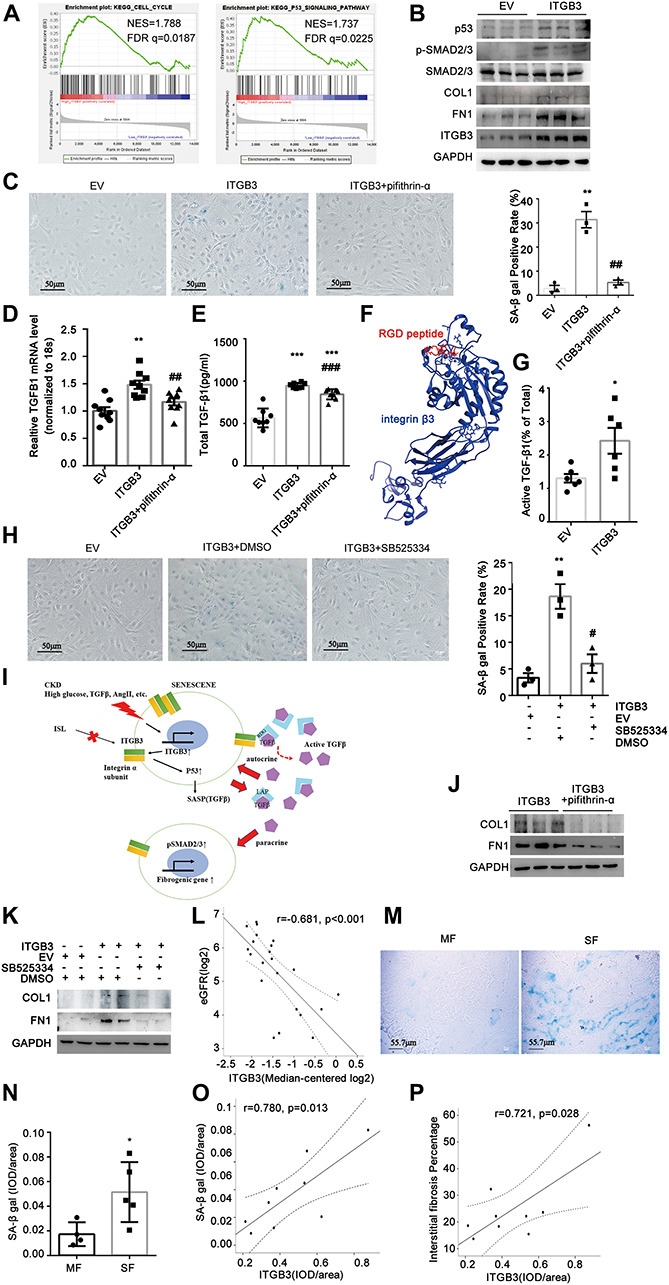
Integrin β3 (ITGB3) aggravated cellular senescence by upregulating p53 signaling pathway and TGF-β secretion. **(A)** GSEA revealed that cell cycle- and p53 signaling pathway-related biological functions were enriched in response to high ITGB3 expression based on the public GSE30529 dataset. **(B)** Expression of ITGB3, p53, SMAD2/3, p-SMAD2/3, COL, and FN1 in cells transfected with ITGB3 or EV. **(C)** Senescence induced upon ITGB3 is shown by an increase in the percentage of cells staining positive for SA-β-gal activity. Senescence induced by ITGB3 could be reversed by pifithrin-α (20 μM, 24 h). Quantification of three independent experiments is shown. **(D)** The mRNA levels of TGFB1 were increased in ITGB3 overexpressed cell. The expression of **(E)** total TGF-β1 and **(G)** ratio of active TGF-β1 in cell medium of HK-2 cells. **(F)** The 3D structure of integrin β3 binding with RGD peptide. **(H)** HK-2 cells were co-cultured with ITGB3-overexpressed or empty vector cells, SB525334 (1 μM, 24 h) could reduce the SA-β Gal staining induced by the conditional medium of ITGB3-overexpressed cells. **(I)** A working model of ITGB3 in senescent signaling in CKD. **(J)** Levels of COL and FN1 protein in ITGB3-overexpressed HK-2 cells treated with pifithrin-α (20 μM, 24 h). **(K)** FN1 and COL1 were upregulated in cells that co-cultured with ITGB3-overexpressed cells compared with EV, and treatment with SB525334 (1 μM, 24 h) could reduce these upregulations. **(L)** Intrarenal ITGB3 mRNA level was significantly associated with eGFR. Representative SA-β-gal **(M)** staining and **(N)** quantification of DN patients with different fibrosis degree. The quantification of ITGB3 IHC staining correlated with **(O)** SA-β-gal staining and **(P)** interstitial fibrosis degree in DN patients (*n* = 9). For statistical analysis, a two-tailed Student’s *t*-test was used for G and N; one-way ANOVA with Tukey’s *post-hoc* test was used for panels **(C–E,H)**; and Pearson association was used for panels **(L,O,P)**. *p < 0.05; **p < 0.01; ***p < 0.001 when compared with EV or MF; #*p* < 0.05; ##*p* < 0.01; ###*p* < 0.001 when compared with ITGB3; EV, empty control. MF, modest fibrosis group. SF, sever fibrosis group.

To investigate the potential role of ITGB3 in CKD, we first performed gene set enrichment analysis (GSEA) based on the GSE30529 dataset. In line with the enrichment in Supplementary Figure 3, this analysis showed significant enrichment of high ITGB3 expression in gene sets related to cell cycle and p53 pathway (Figure 4A), both of which were pivotal in senescence (Lopez-Otin et al., 2013). To evaluate the influence of ITBG3 in tubular cell senescence, we transfected HK-2 cells with an ITGB3-expressing plasmid and confirmed the transfection efficiency by Western blot analysis (Figure 4B). P53 was significantly upregulated (Figure 4B) and *in situ* SA-β-gal staining assay revealed significant augmentation of senescence in ITGB3-overexpressed cells, whereas treatment of pifithrin-α, the inhibitor of p53, could reverse such effects in ITGB3-overexpressed cells (Figure 4C). Taken together, these findings suggest that ITGB3 inducing senescence is at least partially dependent on the p53 pathway.

### Integrin β3 Upregulates Senescence-Associated Secretory Phenotype Associated With Transforming Growth Factor-β

Senescent cells could secrete a complex of factors, referred to as the SASP, which may activate the immune system to eliminate or reinforce senescence (Kuilman and Peeper, 2009; Tchkonia et al., 2013). TGF-β, a crucial molecule in kidney fibrosis, is a non-classic SASP factor in autocrine or paracrine (Tominaga and Suzuki, 2019). Recently, Rapisarda et al. (2017) validated that ITGB3 regulated cellular senescence in a cell-autonomous fashion by activating the TGF-β pathway in human primary cultured fibroblasts. Besides, it is well elucidated that integrins could bind with the RGD peptide in latency-associated peptide (LAP) of latent-TGF-β and promote TGF-β activation (Dong et al., 2017; Campbell et al., 2020). Here, we found ITGB3 could elevate the transcript level of TGF-β1 and the protein level of total TGF-β1; however, when treated with pifithrin-α, these increasement would be attenuated (Figures 4D,E). These results suggested that ITGB3 promoted TGF-β1 expression through induction of cellular senescence. Interestingly, we predicted that ITGB3 interacted with the RGD peptide through molecular simulation (Figure 4F), and ITGB3 could increase active TGF-β1 and activate the TGF-β/SMAD signal pathway (Figures 4B,G). Additionally, because TGF-β1 is a secretory molecule, we investigated whether the senescent cells induced by ITGB3 could mediate other cell senescence *via* paracrine. Thus, we co-cultured HK-2 cells with ITGB3-overexpressed cells or empty vector cells, and treated them with type I TGF-β receptor inhibitor, SB525334, or vehicle control. We found that the conditional medium from ITGB3-overexpressed cells could induce senescence and SB525334 treatment could significantly reduce these effect (Figure 4H). Overall, ITGB3 might induce senescence through TGF-β secretion and activation (Figure 4I).

### Integrin β3 Induction Promotes Kidney Fibrosis via Enhancing Tubular Cell Senescence

Senescent cells are known to play a role in the maladaptive repair, which contributes to progressive renal fibrosis after injury (Xu et al., 2020). To understand whether ITGB3 could induce pro-fibrotic phenotype in HK-2 cells, we examined collagen I (COL1) and FN1 expression in ITGB3-overexpressed HK-2 cells. As Figure 4B shows, ITGB3 overexpression significantly upregulated the expression of COL1 and FN1, while treatment of pifithrin-α could reverse such upregulation (Figure 4J). Besides, co-culturing with ITGB3-overexpressed cells could upregulate COL1 and FN1 expression in HK-2 cells, whereas SB525334 could reduce that, suggesting that ITGB3 might induce renal fibrosis related to senescence (Figure 4K). Furthermore, we found that ITGB3 transcriptional level in kidneys was significantly associated with the baseline eGFR based on Nephroseq database in DN, LN, and FSGS patients, while not in MN and MCD patients (Woroniecka et al., 2011; Berthier et al., 2012; Ju et al., 2015; Figure 4L and Supplementary Figures 4A–D). Then, we observed the ITGB3 expression, senescent status, and renal fibrosis in nine DN patients (Table 1). According to the interstitial fibrosis percentage, we divided them into two groups, the modest fibrosis group and severe fibrosis group. As Figures 4M,N show, SA-β gal was increased in DN patients with more interstitial fibrosis. It was shown that the semiquantitative IHC expression of ITGB3 was significantly associated with SA-β gal staining and renal fibrosis percentage (Figures 4O,P).

**TABLE 1:** Baseline characteristics of DN patients.

	DN
*N*	9
Age (years)	48.79 ± 5.59
Ethnicity	Han
Gender (female, %)	6 (66.7%)
Creatinine (mg/dl)	1.03 ± 0.17
eGFR (ml/min per 1.73 m^2^)	68.99 ± 7.47
Proteinuria (g/24 h)	3.43 ± 1.80
Follow-up (years)	2.53 ± 0.69

*Data are %, or means ± SD.*

### Integrin β3 Overexpression Promotes Renal Fibrosis in Unilateral Ureteral Obstruction Mice

**FIGURE 5:**
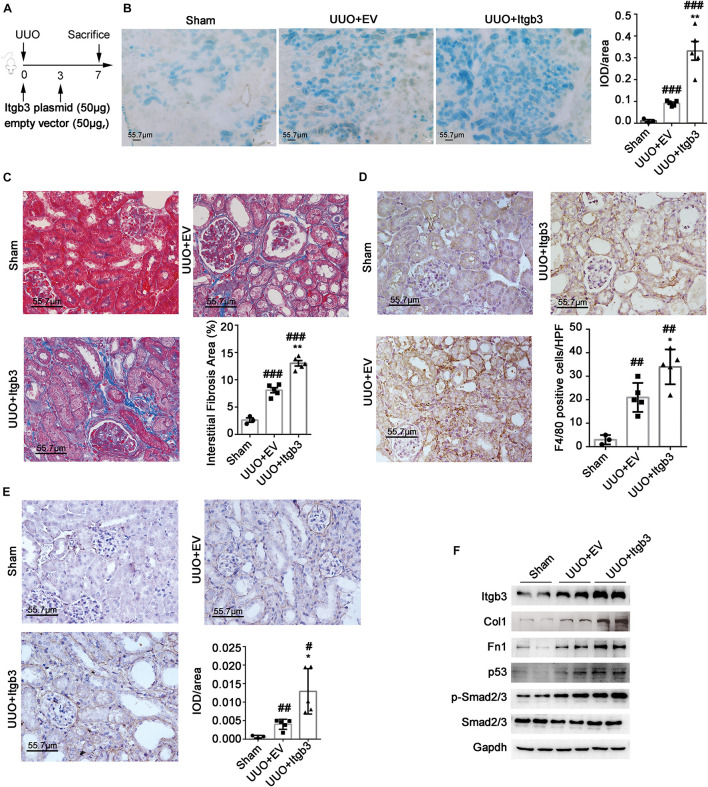
Integrin β3 (ITGB3) overexpression aggravates UUO nephropathy in mice. We injected the Itgb3–expressing plasmids or control plasmids into mice *via* tail injection. **(A)** Schematic diagram of the experimental procedure. Representative images of **(B)** SA-β-gal, **(C)** Masson trichrome, **(D)** F4/80, and **(E)** Fn1 staining of mouse kidneys (day 7 post-UUO, *n* = 5 for UUO + EV and UUO + ITGB3 group, *n* = 3 for Sham group). Right histogram represents quantitative analysis of staining. **(F)** Expression of Itgb3, p53, Smad2/3, pSmad2/3, Col, and Fn1 in renal tubule from UUO mice injected with Itgb3-expressing or EV plasmid and Sham mice. For statistical analysis, a two-tailed Student’s *t*-test was used for panels **(B–E)**. *p < 0.05; **p < 0.01 when compared with the EV group; #*p* < 0.05; ##*p* < 0.01; ###*p* < 0.001 when compared with the Sham group; EV, empty control. Scale bar = 55.7 μm.

To analyze the function of ITGB3 in an animal model, we overexpressed Itgb3 in mouse kidneys *via* delivering Itgb3 plasmid *via* vein-tail injection (Figure 5A). Itgb3 expression was significantly increased in the kidneys of the UUO model, and mainly localized in tubule (Supplementary Figure 5A). As shown in Supplementary Figure 5B, transfection of Itgb3 plasmid strongly increased Itgb3 expression. Next, we investigated the effect of Itgb3 on UUO nephropathy. As shown by Masson trichrome staining and SA-β-gal, F4/80, and Fn1 staining of mouse renal tissues (Figures 5B–E), delivery of Itgb3-expressing plasmid significantly promoted the tubulointerstitial fibrosis, the infiltration of inflammatory cells, and cellular senescence after UUO. Besides, along with the results in cultured cells, Itgb3 induction increased the activity of p53 and pSmad2/3, and the expression of Col1 and Fn1 (Figure 5F).

### Isoliquiritigenin Is a Candidate Drug to Suppress Tubular Cell Senescence

**FIGURE 6:**
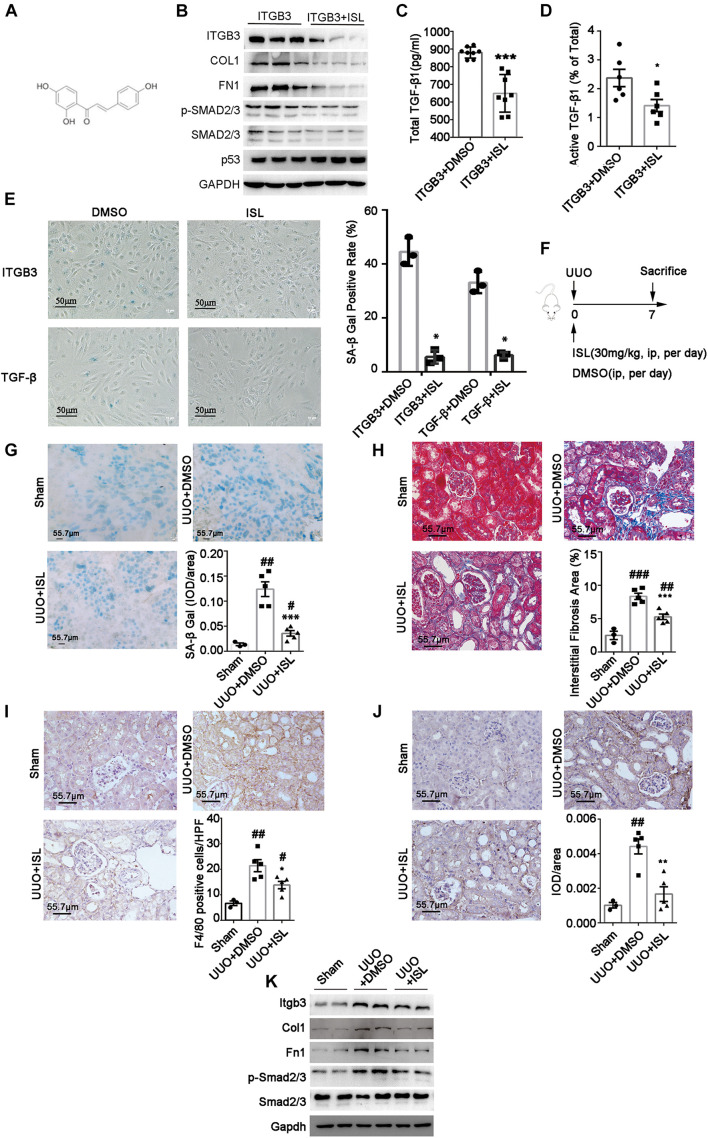
Isoliquiritigenin (ISL) attenuates ITGB3 expression and renal fibrosis in UUO mice. **(A)** The structure of ISL. **(B)** Expression of Itgb3, p53, Smad2/3, pSmad2/3, Col, and Fn1 in ITGB3-overexpressed HK-2 cells treated with DMSO or ISL (20 μM, 24 h). ELISA for **(C)** total and **(D)** active TGF-β1 in culture medium of HK-2 cells transfected with EV or ITGB3 plasmid. **(E)** SA-β-gal staining of ITGB3-overexpressed HK-2 cells treated with DMSO or ISL (20 μM) for 24 h (the upper one), or HK-2 cell stimulated with TGF-β (10 ng/ml) for 24 h, then treated with DMSO or ISL (20 μM) for 24 h (the below one). **(F)** Schematic diagram of the experimental procedure. **(G)** SA-β-gal staining in frozen sections of UUO mice treated with DMSO or ISL and Sham mice (*n* = 5 for UUO + EV and UUO + ITGB3 group, *n* = 3 for Sham group). **(H)** MASSON staining of UUO mice treated with DMSO or ISL (*n* = 5 per group). IHC analysis of **(I)** F4/80 and **(J)** Fn1 expression in the renal tissues of UUO mice treated with DMSO or ISL and Sham mice (*n* = 5 for UUO + EV and UUO + ITGB3 group, *n* = 3 for Sham group). **(K)** Western blot analysis of Itgb3, Col, Fn1, Smad2/3, and pSmad2/3 in tubular tissues of UUO mice treated with DMSO or ISL and Sham mice (*n* = 5 for UUO + EV and UUO + ITGB3 group, *n* = 3 for Sham group). For statistical analysis, a two-tailed Student’s *t*-test was used for panels **(C–E,G–J)**. *p < 0.05; **p < 0.01; ***p < 0.001 when compared with the DMSO group; #*p* < 0.05; ##*p* < 0.01; ###*p* < 0.001 when compared with the Sham group. Scale bar = 55.7 μm, unless otherwise indicated.

To explore a chemical compound to target ITGB3, we used CMAP, an approach that has been used widely in cancer drug discovery, to screen a candidate drug (Peng et al., 2015). As Table 2 shows, we screened ISL as the candidate agent to antagonize the effect induced by ITGB3 based on the GSE30529 dataset (for the full data of Table 2 and Supplementary Data 2). ISL is a chalcone, which is characterized by its antioxidant and anti-inflammatory properties (Figure 6A). Herein, we investigated the protective effect of ISL in HK-2 cells against ITGB3-induced senescence. It was found that ISL treatment attenuated ITGB3-induced TGF-β pathway activation, TGF-β secretion and activation, SA-β-Gal staining, and pro-fibrotic phenotypic change, but without change in the expression level of p53 (Figures 6B–E). Furthermore, ISL could inhibit TGF-β-induced tubular cell senescence (Figure 6E). These data suggest that targeted ITGB3 with ISL could reverse tubular senescence and pro-fibrotic phenotypic change.

**TABLE 2:** The top 10 candidate agents that could inhibit the effect of ITGB3.

Score	Name	Description
−97.51	Diethylstilbestrol	Estrogen receptor agonist
−94.47	ZK-756326	CC chemokine receptor ligand
−93.02	Isoliquiritigenin	Guanylate cyclase activator
−92.26	Afatinib	EGFR inhibitor
−90.99	Panobinostat	HDAC inhibitor
−90.67	MEK1-2-inhibitor	MEK inhibitor
−89.91	BNTX	Opioid receptor antagonist
−89.22	6-aminochrysene	Transferase inhibitor
−89.21	NVP-AUY922	HSP inhibitor
−88.31	Zalcitabine	Nucleoside reverse transcriptase inhibitor

### Isoliquiritigenin Treatment Ameliorates Unilateral Ureteral Obstruction Nephropathy in Mice

To confirm the effect of ISL in renal injury, we established UUO model and treated them with ISL (Figure 6F). The ISL-treated group showed a significant reduction of renal fibrosis, inflammatory cell infiltration, and SA-β-gal staining compared with the disease control (Figures 6G–J). Additionally, the protein abundance of Itgb3, p-SMAD2/3, Col1, and Fn1 were also significantly decreased in the ISL-treated group (Figure 6K). These findings indicate that ISL might be used as a therapeutic agent to target tubular cell senescence and kidney fibrosis.

## Discussion

Tubular epithelial cells are frequently implicated in renal senescence to induce renal injury and fibrosis *via* p53 or p21 signal pathway in kidney (Sis et al., 2007). Previous studies about tubular senescence were mainly focused on the intracellular processes, while the extracellular signal delivery of senescence was rarely touched in kidney. ITGB3 is a multifunctional cell surface receptor that mediates many downstream signals, including senescent signal (Hynes, 2002; Rapisarda et al., 2017). Here, we identified a mechanistic link between integrin signaling and senescence during kidney injury. Moreover, we found that targeting ITGB3 with ISL could protect against tubular cell senescence and kidney fibrosis.

Both the stable cell cycle arrest and p53 pathway activation are the classical features of cellular senescence (Hernandez-Segura et al., 2018). The continuous accumulation of senescent cells leads to the age-related deterioration of vital organs and thus constitutes an organism’s aging process. Recent studies showed that CKD presents as a clinical model of premature aging (Xu et al., 2020). Notably, hyperglycemia could directly induce cellular senescence in mesangial and tubular cells (del Nogal et al., 2014). p53, p16, and SA-β-gal abundance was increased in renal tubular cells under high glucose conditions (Verzola et al., 2008). Moreover, the p21cip1/p27kip1 knockout mouse showed reduced proteinuria, glomerular hypertrophy, and tubule-interstitial damage in a DN model (Al-Douahji et al., 1999; Wolf et al., 2005).

Integrins are multifunctional heterodimeric cell surface receptor molecules that mediate downstream intracellular signals. Integrin signaling affects numerous cellular processes, including cell adhesion, migration, proliferation, survival, and differentiation (Seguin et al., 2015). Interestingly, integrins can also regulate senescence (Rapisarda et al., 2017). Here, we provided the evidence that treatment with the p53 inhibitor, pifithrin-α, prevented the senescent and profibrotic phenotype induced by ITGB3 in HK-2 cells.

Senescent cells secrete an array of inflammatory molecules, growth factors, and metalloproteases that collectively constitute the SASP. SASP components include IL-1b, IL-6, IL-8, and TGF-β1 (Tominaga and Suzuki, 2019). These factors may drive chronic inflammation and senescence and they could affect the neighboring cells and further exacerbate their regenerative capacity in a paracrine and autocrine fashion (Coppe et al., 2010; Davalos et al., 2010; He and Sharpless, 2017). SASP is associated with many age-related diseases (Watanabe et al., 2017). We observed that overexpression of ITGB3 could increase the secretion and activation of TGF-β in tubular cells, and treatment with ISL could reverse the increasement.

Transforming growth factor-β, an important and classic factor in modulating inflammation and fibrosis, is synthesized as an inactive latent precursor that requires cleavage and/or dissociation from the LAP to engage the TGF-βR complex (Travis and Sheppard, 2014). Integrins are a family of heterodimeric cell surface receptors consisting of α and β subunits, and 27 total integrin subunits (19 α and 8 β) have been identified to date (Takagi and Springer, 2002). Among the integrins, five share the a subunit (αvβ1, αvβ3, αvβ5, αvβ6, and αvβ8) and are capable of binding the RGD tripeptide sequence on the LAP of TGF-β (Weaver et al., 2007; Pociask and Kolls, 2010). The αv integrins are important physiological regulators of TGF-β activation; for example, latent-TGF-β binding to αvβ6 triggers a global conformational change from extended closed to extended open, allowing actin cytoskeletal force to be transmitted through the β subunit to release mature TGF-β from its latent complex (Dong et al., 2017). It is reasonable to speculate that the overexpression of ITBG3 promotes the physical binding between αvβ3 and LAP, and augments the activation of TGF-β. Through protein–peptide docking, we simulated that RGD peptide could dock to ITGB3, and in an *in vitro* study, we observed that the overexpression of ITGB3 could induce the secretion of active TGF-β in cell medium, and conditional medium of ITGB3-overexpressed cells could induce senescent and profibrotic phenotype *via* paracrine, but the detailed structure and process still need be further explored.

Isoliquiritigenin is a bioactive ingredient isolated from the roots of plants belonging to licorice family. The previous research demonstrated that licorice could ameliorate nephrotoxicity in acute renal tubular necrosis (Aksoy et al., 2012). However, the active components from licorice still need to be explored. As an extract of licorice, ISL is applied on various disease prevention or treatment, such as anti-cancer therapy, antibiotic therapy, anti-oxidative therapy, anti-inflammation therapy, and so forth (Peng et al., 2015). Previously, studies had shown that ISL could inhibit inflammation involving NF-κB pathway to protect kidney from lesions in various models, such as acute kidney injury, DN, hypertensive renal injury, and UUO-induced renal fibrosis (Lee et al., 2013; Xiong et al., 2018; Liao et al., 2020). Li et al. (2010) demonstrated that ISL could block TGF-β/SMAD signaling and retard high glucose-induced mesangial matrix accumulation. Based on the CMAP database, we screened ISL as the ITGB3-targeted agent, and in an *in vitro* study, we found that the ISL treatment could suppress the expression of ITGB3 and improve the aging phenotype induced by TGF-β or ITGB3, such as SA-β-gal staining and TGF-β secretion. Surprisingly, treatment of HK-2 cells with ISL could not reverse the upregulation of p53 protein levels, suggesting that ISL inhibits senescence independent of p53. As senescent cells are accumulated during aging, causing chronic inflammation and kidney injury, ITGB3 could be a potential therapeutic route to block inflammation and kidney injury and fibrosis.

There are some limitations in our study. The first one is that the roles of ISL in retarding senescence and fibrosis were mainly observed in mouse models and cultured cells but not in patients. Although we did detect the upregulation of ITGB3 and its positive correlation with the degree of kidney senescence and fibrosis in DN patients, the effectiveness and importance of targeting ITGB3 in protecting against senescence remain to be further explored by a clinical trial in the future. The second one is that we did not utilize Itgb3 knockout mice in our study. Although we did interfere with the expression of Itgb3 through plasmid transfection *via* vein-tail injection, the research in transgenic mice are still needed to carry out in the future.

In conclusion, this study demonstrated that the upregulation of ITGB3 markedly accelerates renal fibrosis possibly by increasing cellular senescence *via* p53 pathway, TGF-β associated SASP, and TGF-β activation. Additionally, we identified ISL as an effective agent to antagonize ITGB3 and ameliorate senescence and fibrosis. These findings from the current research not only increase our understanding of the pathogenesis of senescence in kidney but also provide therapeutic potential of ISL or other ITGB3 inhibitors. Performing clinical trials of ISL in patients with CKD such as DN may offer a viable approach for the therapy.

## Data Availability Statement

The original contributions presented in the study are included in the article/Supplementary Material, further inquiries can be directed to the corresponding author/s.

## Ethics Statement

The studies involving human participants were reviewed and approved by National Clinical Research Center of Kidney Diseases, Jinling Hospital, Nanjing University Medical School, Nanjing, China. The patients/participants provided their written informed consent to participate in this study. The animal study was reviewed and approved by National Clinical Research Center of Kidney Diseases, Jinling Hospital, Nanjing University Medical School, Nanjing, China. Written informed consent was obtained from the individual(s) for the publication of any potentially identifiable images or data included in this article.

## Author Contributions

SL conceived the study and wrote the manuscript. SL, SJ, QZ, BJ, and WL performed the experiments. DL established the PHN model. SL and MZ analyzed the transcriptome data. CJ, CD, and ZL revised the manuscript. All authors reviewed the manuscript.

## Conflict of Interest

The authors declare that the research was conducted in the absence of any commercial or financial relationships that could be construed as a potential conflict of interest.

## Publisher’s Note

All claims expressed in this article are solely those of the authors and do not necessarily represent those of their affiliated organizations, or those of the publisher, the editors and the reviewers. Any product that may be evaluated in this article, or claim that may be made by its manufacturer, is not guaranteed or endorsed by the publisher.
