# Supplementary material for: Integrin β3 Induction Promotes Tubular Cell Senescence and Kidney Fibrosis
Source: Front Cell Dev Biol. 2021 Nov 5;9:733831. doi: 10.3389/fcell.2021.733831 (PMC8602096; doi:10.3389/fcell.2021.733831)
Supplement: Supplementary file 3 [file Data_Sheet_3.DOCX]

Supplementary materials

Methods

Data collection

The GSE30529 and GSE118089 datasets were downloaded from the Gene Expression Omnibus (GEO, <https://www.ncbi.nlm.nih.gov/geo/>) database. The GSE30529 dataset contains gene expression profiles of 13 patients with DN and 12 healthy controls. The GSE118089 dataset contains gene expression profiles of 6 STZ mice and 6 vector controls.

Identification of differentially expressed genes (DEGs)

We used the limma R package to investigate differentially expressed genes (DEGs). By controlling the false discovery rate (FDR), we define DEGs as genes with adj. P-value <0.05 and |log2 fold change (FC)|>1.

Functional and pathway enrichment analysis

Functional and pathway enrichment analyses were performed for DEGs using the clusterProfiler R package, including Gene Ontology (GO) terms and Kyoto Encyclopedia of Genes and Genomes (KEGG) pathway enrichment analyses. GO terms

and KEGG pathways with an adj. P-value <0.05 were considered significant.

Hub gene identification

The PPI network was retrieved from the Search Tool for the Retrieval of Interacting Genes (STRING, http://www.string-db.org/). MCODE plugin in Cytoscape was used to identify the most significant module and hub gene in the network[^1^](#_ENREF_1).

Gene set enrichment analysis (GSEA)

The GSEA software (version 3.0, www.broadinstitute.org/gsea/) was used to identify gene signatures between groups with high (top 50%, n = 13) and low (bottom 50%, n = 12) ITGB3 expression based on the GSE30529 dataset. The results of GSEA are expressed using normalized enrichment scores (NES) that take into account the size and degree of over-representation of the gene set at the top or bottom of the aligned gene list (P < 0.05 and false discovery rate (FDR) ≤ 0.25).

Characteristics of Study Populations.

DN patients are diagnosed by renal biopsy and all signed informed consent.

Isolation of rodent tubule

Mouse tubule were isolated by using the magnetic bead-based isolation technique as previously described[^23^](#_ENREF_23)^,^ [^24^](#_ENREF_24). Briefly, mice were anesthetized and perfused with bead solution (Dynabead M450, Invitrogen). The kidneys were then removed, minced into 1mm^3^ pieces, and digested in digestion solution buffer (300 U/ml collagenase type II, 1 mg/ml proteinase E, and 50 U/ml DNase I) for 15 min at 37°C. After digestion, tissues were pressed gently through a 100µm strainer and absorbed by magnetic particle concentrator. The supernatant was collected and then passed through a 35mm cell strainer. The tubule tissues remaining on the cell mesh were rinsed with cold PBS and collected by centrifugation.

RNA extraction, reverse transcription, and real-time RT-PCR

Total RNA from cell lines and freshly-frozen tissues was isolated by TaKaRa MiniBEST Universal RNA Extraction Kit (TaKaRa). Reverse transcription was performed using PrimeScript™ RT Master Mix (TaKaRa). The SYBR® Premix Ex Taq™ II (TaKaRa) were used in quantitative RT-PCR analysis. GAPDH or 18S were used as endogenous controls for normalization. All primer sequences are listed in Supplementary Table S2.

Western blot analysis

Western blot assay was performed as previously described[^25^](#_ENREF_25). As primary antibodies, anti-ITGB3 (Santa Cruz), anti-P53 (Beyotime Biotechnology), anti-SMAD2/3 (Sigma-Aldrich), anti-p-SMAD2/3 (Sigma-Aldrich), anti-COL1 (Santa Cruz), anti-FN1 (Proteintech) and anti-GAPDH (Bioworld) were used.


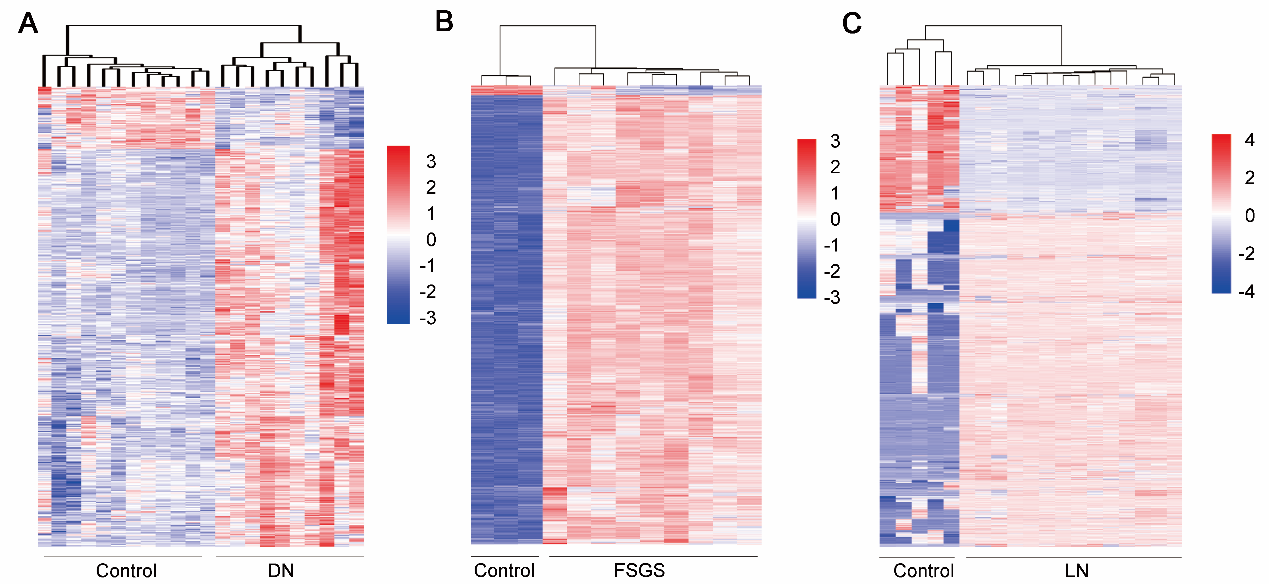


Fig.S1 Heatmap of DEGs. The heatmap of DEGs in (A) DN, (B) FSGS, (C) LN mice.


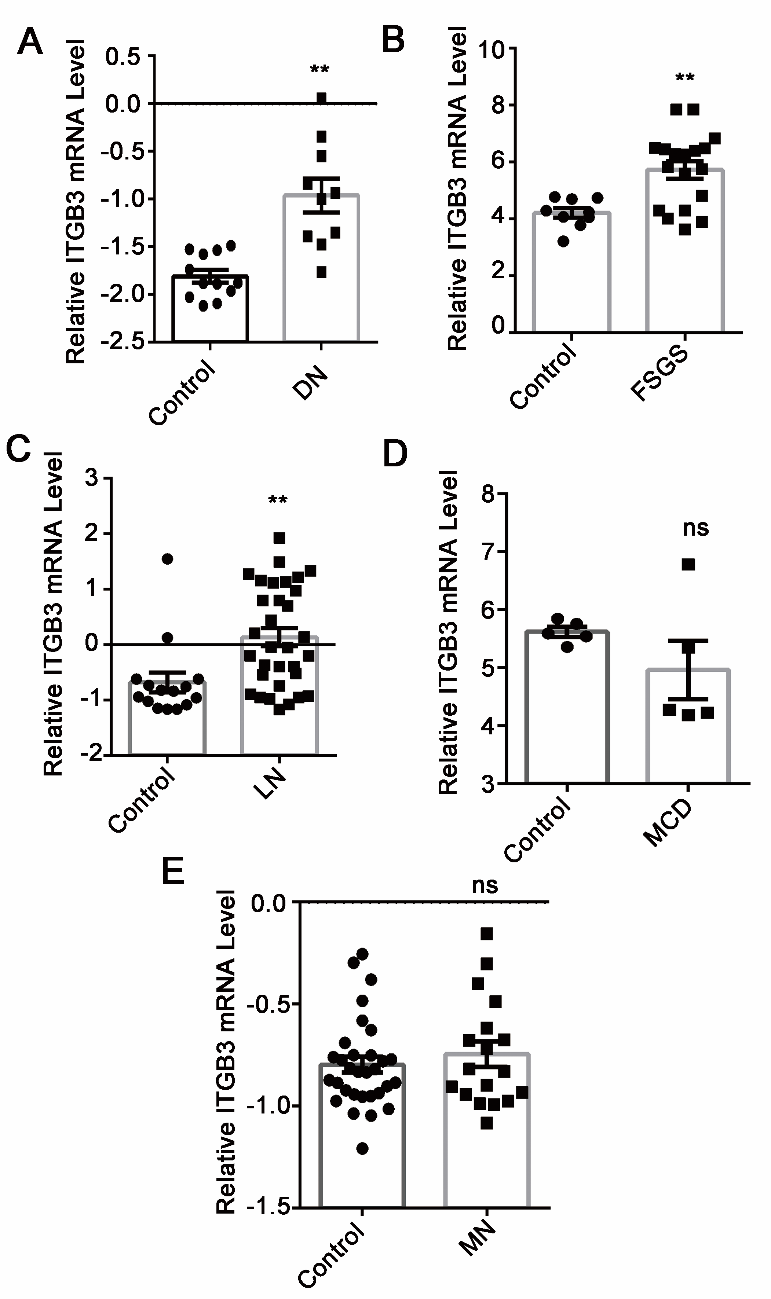


Fig.S2 Expression of ITGB3 in tubulointerstitial tissues of FSGS, LN, MCD and MN patients. The mRNA expression of ITGB3 in tubulointerstitial tissues of patients with

(A) DN (Control=12, DN=10), (B) FSGS (Control=9, FSGS=18), (C) LN (Control=15, LN=32), (D) MCD (Control=5, MCD=5) and (E) MN (Control=31, MN=18). For statistical analysis, a two-tailed Student’s t test was used for A-E. **, P < 0.01. ns= no significance.


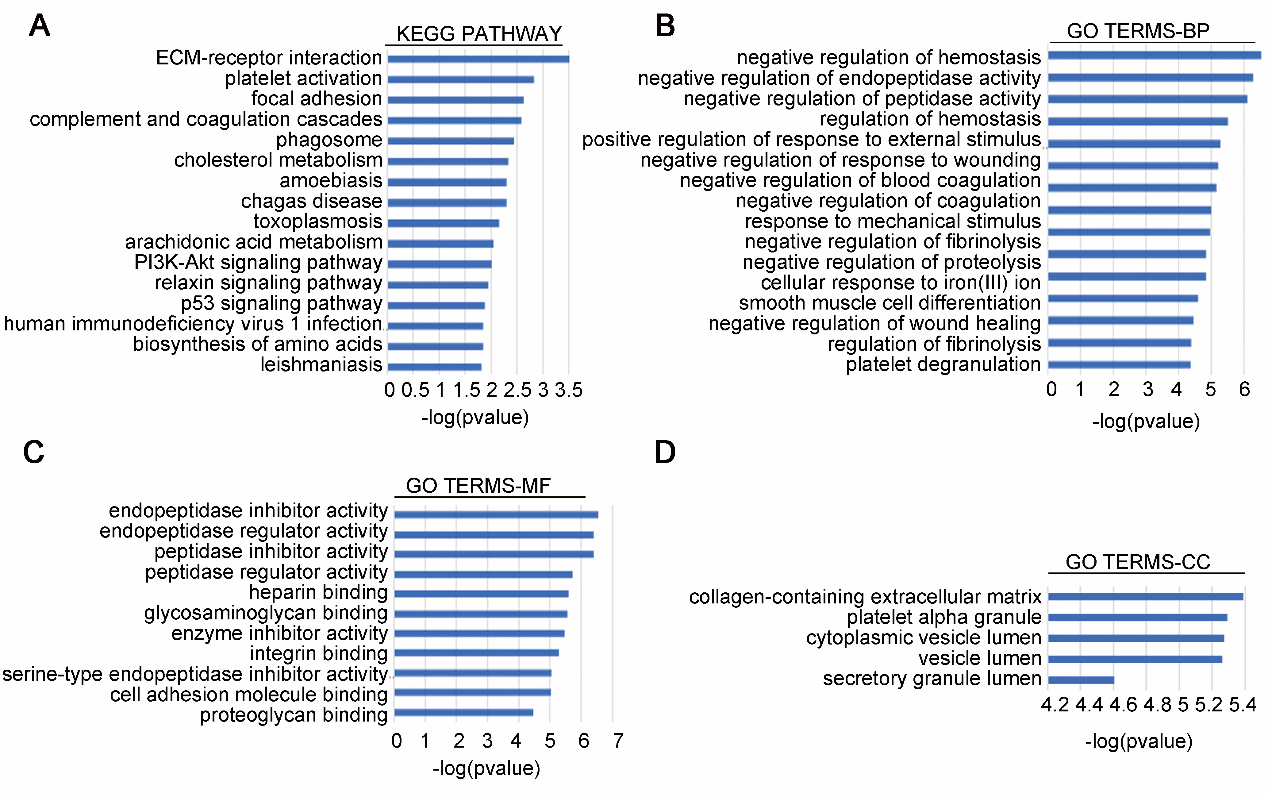


Fig.S3 Significantly enriched GO terms and KEGG pathways of DEGs. DEGs functional and signaling pathway enrichment was conducted by KEGG (A), GO-terms BP (B), MF (C) and CC (D). MF: molecular function, BP: biological process and CC: cellular component.


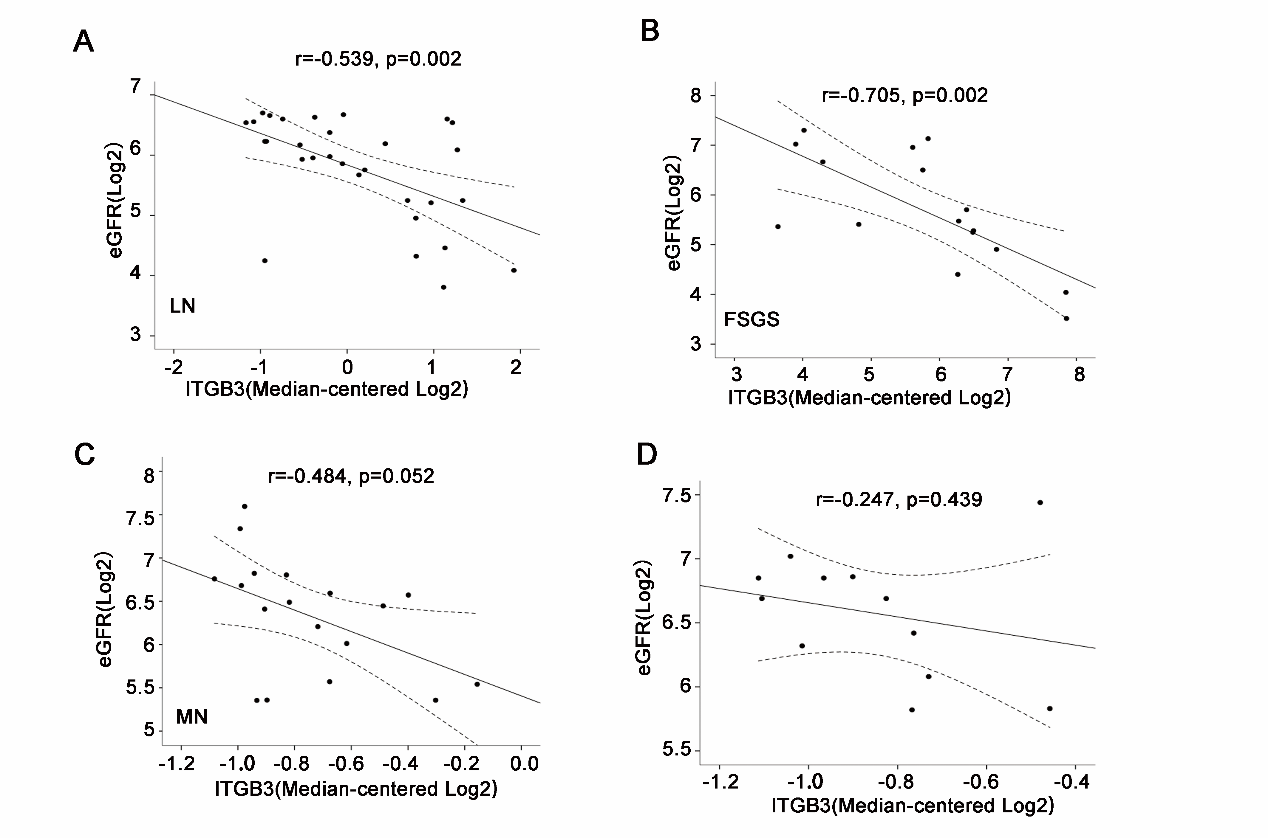


Fig.S4 The association of ITGB3 with baseline eGFR in LN, FSGS, MN and MCD patients. The mRNA expression of ITGB3 was significantly associated with the baseline eGFR in of patients with (A) LN (n=30) and (B) FSGS (n=16). There was no correlation between the mRNA level and eGFR in patients with no significant tubular injury, such as (C) MN (n=18) and (D) MCD (n=12). For statistical analysis, Pearson association was used for A-D.


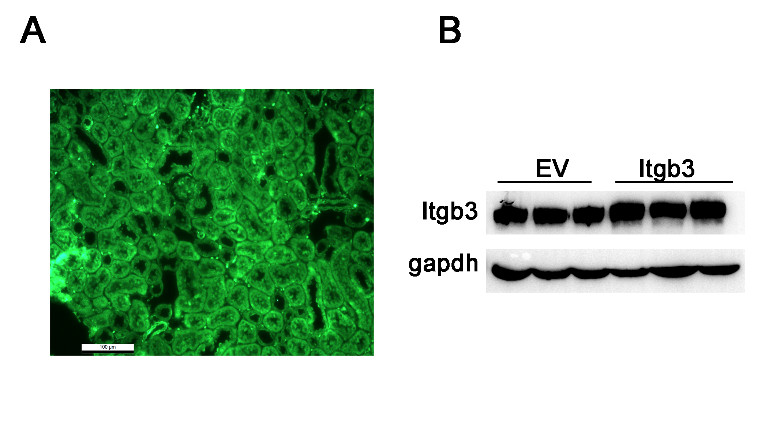


Fig.S5 The expression of Itgb3 in UUO mice injected with Itgb3-expressing plasmid. (A) overexpressed Itgb3 mainly localized in tubule. (B) Itgb3 significantly increased in the renal tubule from UUO mice injected with Itgb3-expressing plasmid.

Table S1. Top 10 DEGs in string interaction.

| ID | INTERACTION |
| --- | --- |
| FN1 | 42 |
| CD44 | 36 |
| PTPRC | 34 |
| ITGB3 | 22 |
| VWF | 22 |
| LCP2 | 22 |
| SELL | 21 |
| PECAM1 | 21 |
| COLA2 | 20 |
| ITGAV | 20 |

Table S2. Primers.

| Human-ITGB3-F | TCCCTCATCCATAGCACCTCC |
| --- | --- |
| Human-ITGB3-R | CCTATGCTCTCCTTCTTTGCCAT |
| Mouse-Itgb3-F | GATCGCTGTCAAAACACCTGA |
| Mouse-Itgb3-R | GCCTGTAATTGTAGCACCTGA |
| Human-TGFB1-F | TACCTGAACCCGTGTTGCTCT |
| Human-TGFB1-R | AACCCGTTGATGTCCACTTGC |
| 18S-F | TTTCTCGATTCCGTGGGTGG |
| 18S-R | AGCATGCCAGAGTCTCGTTC |
| Gapdh-F | CCATCTTCCAGGAGCGAGAC |
| Gapdh-R | TTTCTCGTGGTTCACACCCAT |

REFERENCES:

1. Shannon P, Markiel A, Ozier O*, et al.* Cytoscape: a software environment for integrated models of biomolecular interaction networks. *Genome Res* 2003; **13:** 2498-2504.

2. Wu J, Zheng C, Wang X*, et al.* MicroRNA-30 family members regulate calcium/calcineurin signaling in podocytes. *J Clin Invest* 2015; **125:** 4091-4106.

3. Yuqiu L, Yuting Y, Qianqian Y*, et al.* Single-cell RNA-sequence analysis of mouse glomerular mesangial cells uncovers mesangial cell essential genes. *Kidney Int* 2017; **92**.

4. Difei Z, Yuexian X, Wenju L*, et al.* Renal tubules transcriptome reveals metabolic maladaption during the progression of ischemia-induced acute kidney injury. *Biochem Biophys Res Commun* 2018.
